# A Method of Expressing Ear-Power in Degrees of a Fixed Scale

**Published:** 1885-09

**Authors:** R. A. Chudleigh

**Affiliations:** Wimborne, Dorset


### A METHOD OF EXPRESSING EAR-POWER IN DEGREES OF A FIXED SCALE.

BY

THE REV. R. A. CHUDLEIGH, M.A.,

*Wimborne, Dorset.*

---

SCIENCE becomes exact and trustworthy in proportion as it deals with number, measure and weight. Whatever is vague and ill-defined must be replaced by what is definite and numerical. Prescriptions, for instance, which used to order "a pinch" of one ingredient, "a little" of another, and "a good drop" of a third, are now given in drams and scruples. Visual capacity has for some years been gauged by accurate tests; and if the total visual power be defective, it can be so analysed as to localise the defect either in the horizontal plane, or in the vertical, or in some other direction. But the tests for hearing seem to be still vague and indefinite. A watch is placed on different parts of the head, and the patient informs us that he can hear the ticking "moderately," or "only just," or "fairly well," or "scarcely at all." One would like to reduce these hazy expressions to feet and inches, and represent them in terms of the table called Long Measure. A very simple apparatus, often used as a toy for amusement, suggests a method, not only of representing one's total ear-power numerically, with reference to a fixed standard, but also, in cases of defect, of so separating the total ear-power into its two components (air-conduction and bone-conduction) as to show approximately what proportion of the defect is due to obstruction in the meatus, and what proportion is due to disorganisation of the labyrinth or degeneration of the eighth nerve. Take a yard and a half of whipcord, or any tight twist. Let a person of normal hearing place his open hands flat on his ears, cupping the hands a little, so as to make over each ear a hollow as nearly as possible air-tight. Tie one end of the cord around the horizontal circumference of the head, including the hands, or (far better) use a slip-knot, and run it up tight to the forehead, so arranging matters that the knot shall rest on the region of the frontal sinus, and the cord shall cross the hands near the distal ends of the metacarpals. Now take the free end of the cord in your left hand and strain it tight from the wearer's forehead, so as to keep it in tension, and with the thumb-nail of your right hand briskly scratch the strained cord, making sudden starts and long draws of the nail alternately. If this be fairly well managed, the result to the wearer of the cord will be a wonderfully good imitation of a thunder-peal, while to a bystander the friction on the cord is almost inaudible.

Having succeeded, in the first place, in making this very simple experiment answer, it will next occur to one to ascertain at how great a distance on a long cord a scratch is discernible. If one's friends be induced to submit to experiment, it soon becomes apparent that to different persons a scratch is audible at very different distances, according to their varying ear-power. Differences of hearing so slight as to have escaped notice will make a difference of ten or fifteen feet on the cord; and before long one gets one's acquaintance distributed, according to their ears, over nearly a hundred yards of whipcord. But the scratch is too loud for a convenient test, and the length of cord required is unwieldly. The arrangement which I have found most suitable is this: Take fifty feet of whipcord; mark it off into lengths of six inches, which is readily done by winding it on to a card of the proper breadth (not quite six inches), and chalking or otherwise colouring the two edges. The cord, thus divided into a hundred equal parts, is stretched from one end of a long room or passage to a chair at the other end, on which the subject of experiment is to sit. This end must be provided with a loop (over and above the fifty feet), to fit around the head as before. The test-sound is made by striking the cord with a stiff feather, and the operator notes in what six-inch division the sound ceases to be audible to the person in the chair. A very defective ear may cease to discern anything at perhaps the eighth division. Cases of exceptional acuteness may reach beyond the scale altogether, even to the 126th half-foot; but most persons find their limit somewhere between the fortieth and fiftieth foot—that is, between 80 and 100 of the scale; and as the scale is a centigrade, these figures may be conveniently represented in decimal form as ·08 and 1·26 and ·80 or 1·00 respectively.

It is clear that if the full scale of one hundred hundredths (unity) be taken as perfect hearing, and a given subject ceases to hear the test at the seventy-fifth division, that subject may be said to possess seventy-five hundredths, or three-quarters, of perfect ear-power; and I describe that condition by saying that ·75 is the said person's aural co-efficient. This co-efficient I briefly denote by the symbol C.

It follows from the nature of the experiment that C represents the total hearing power through all channels, whether bone or air. But in cases of defective hearing, it is a great object to ascertain how much of the total capacity is due to air-conduction and how much to bone-conduction. If we call air-conduction A, and bone-conduction B, we may say that $A + B = C$; and the thing required is to find the ratio of A to B. This can be done approximately, though not absolutely. Returning to the cord, remove the loop from the horizontal circumference and lay it on the sagittal suture (the hair should be parted in the middle for the purpose), stretching it over as much as possible of the dome of the head from glabella to occiput, and ascertain as before the point on the scale at which the test-sound ceases to be heard. The distance, which will be much less than when the cord is in the other position, may be fairly taken as a measure of the power of hearing through bone-conduction alone, consequently all that remains (of C) must be due to air-conduction; and, of course, the value of A can be readily found by subtracting B from C, and then A, B and C will be known quantities. But, as before stated, our knowledge of A and B is approximate, not absolute; for it must not be assumed that the bone-conduction in the second experiment is equal to, or identical with, that which formed part of C in the first experiment, because the bones involved in the two cases are very different. It *is*, however, assumed that the one stands to the other in an approximately fixed ratio; so that when we subtract B from C, though we may not subtract the precise quantity of C which is due to bone-conduction, yet we do subtract a quantity which bears to the true quantity a fairly constant proportion.

I will conclude with two concrete examples of how this ear-gauge may be used. Having satisfied oneself by trial on a sufficient number of persons that total ear-power (C) normally equals 1·00, and that hearing per bone alone (B) normally equals, say, ·45, and that therefore hearing per air alone (A) normally equals ·55, we will find the value of A, B and C for the first of our two patients. C is found to be ·72. It is much below normal, and the question is, Where is the defect? B turns out to be ·43, only two degrees below perfection. We at once decide that the auditory nerve is probably sound, but that there is an obstruction somewhere in the *approaches;* it may be that the meatus wants syringing, or a cold has temporarily thickened the walls of the eustachian tube or tympanum. But in the second patient, though we find that the value of C is again ·72, yet B, instead of being nearly normal, proves to be only ·32; whence we conclude that the approaches may be clean and clear enough, but that there is a defect in the eighth nerve or its immediate labyrinthine connections.

